# Supplementary material for: A fault-intrusion-tolerant system and deadline-aware algorithm for scheduling scientific workflow in the cloud
Source: PeerJ Comput Sci. 2021 Nov 2;7:e747. doi: 10.7717/peerj-cs.747 (PMC8576554; doi:10.7717/peerj-cs.747)
Supplement: Supplemental Information 6 [file peerj-cs-07-747-s006.zip › Myproject1.1/docs/org/workflowsim/WorkflowSimTags.html]

WorkflowSimTags


JavaScript is disabled on your browser.


- Overview
- Package
- Class
- Use
- Tree
- Deprecated
- Index
- Help

- Prev Class
- Next Class

- Frames
- No Frames

- All Classes

- Summary:
- Nested |
- Field |
- Constr |
- Method

- Detail:
- Field |
- Constr |
- Method


org.workflowsim

## Class WorkflowSimTags

- java.lang.Object
- - org.workflowsim.WorkflowSimTags

- ---

    

  ```
  public class WorkflowSimTags
  extends java.lang.Object
  ```

  This WorkflowSimTags include tags that are not supported in CloudSimTags

  Since:
  :   WorkflowSim Toolkit 1.0

- - ### Field Summary

    Fields

    | Modifier and Type | Field and Description |
    | --- | --- |
    | `static int` | `CLOUDLET_CHECK` |
    | `static int` | `CLOUDLET_UPDATE` |
    | `static int` | `JOB_SUBMIT` |
    | `static int` | `START_SIMULATION` |
    | `static int` | `VM_STATUS_BUSY` VM Status is busy (no jobs should run on this vm) |
    | `static int` | `VM_STATUS_IDLE` VM Status is idle (a job can run on this vm) |
    | `static int` | `VM_STATUS_READY` VM Status is ready (not used) |
  - ### Method Summary

    - ### Methods inherited from class java.lang.Object

      `clone, equals, finalize, getClass, hashCode, notify, notifyAll, toString, wait, wait, wait`

- - ### Field Detail


    - #### VM\_STATUS\_READY

      ```
      public static final int VM_STATUS_READY
      ```

      VM Status is ready (not used)

      See Also:
      :   Constant Field Values


    - #### VM\_STATUS\_BUSY

      ```
      public static final int VM_STATUS_BUSY
      ```

      VM Status is busy (no jobs should run on this vm)

      See Also:
      :   Constant Field Values


    - #### VM\_STATUS\_IDLE

      ```
      public static final int VM_STATUS_IDLE
      ```

      VM Status is idle (a job can run on this vm)

      See Also:
      :   Constant Field Values


    - #### START\_SIMULATION

      ```
      public static final int START_SIMULATION
      ```

      See Also:
      :   Constant Field Values


    - #### JOB\_SUBMIT

      ```
      public static final int JOB_SUBMIT
      ```

      See Also:
      :   Constant Field Values


    - #### CLOUDLET\_UPDATE

      ```
      public static final int CLOUDLET_UPDATE
      ```

      See Also:
      :   Constant Field Values


    - #### CLOUDLET\_CHECK

      ```
      public static final int CLOUDLET_CHECK
      ```

      See Also:
      :   Constant Field Values


- Overview
- Package
- Class
- Use
- Tree
- Deprecated
- Index
- Help

- Prev Class
- Next Class

- Frames
- No Frames

- All Classes

- Summary:
- Nested |
- Field |
- Constr |
- Method

- Detail:
- Field |
- Constr |
- Method
